# Comparative analysis of two genomes of *Chlamydia pecorum* isolates from an Alpine chamois and a water buffalo

**DOI:** 10.1186/s12864-022-08860-7

**Published:** 2022-09-10

**Authors:** Sara Rigamonti, Anna Maria Floriano, Erika Scaltriti, David Longbottom, Morag Livingstone, Francesco Comandatore, Stefano Pongolini, Lorenzo Capucci, Maria Lucia Mandola, Moira Bazzucchi, Paola Prati, Nadia Vicari

**Affiliations:** 1 National Reference Laboratory for Chlamydioses, Istituto Zooprofilattico Sperimentale della Lombardia e dell’Emilia Romagna (IZSLER), Pavia, Italy; 2 Faculty of Science, University of South Bohemia, České Budějovice, Czech Republic; 3 Risk Analysis and Genomic Epidemiology Unit, Istituto Zooprofilattico Sperimentale della Lombardia e dell’Emilia Romagna (IZSLER), Parma, Italy; 4 Moredun Research Institute, Pentlands Science Park, Bush Loan, Edinburgh, Scotland UK; 5 Sky Net UNIMI Platform–Pediatric Clinical Research Center Romeo ed Enrica Invernizzi, “L. Sacco” Department of Biomedical and Clinical Sciences, University of Milan, Milan, Italy; 6 Proteomics Unit, Istituto Zooprofilattico Sperimentale della Lombardia e dell’Emilia Romagna (IZSLER), Brescia, Italy; 7 Specialist Diagnostic Virology Unit, Istituto Zooprofilattico Sperimentale del Piemonte, Liguria e Valle d’Aosta (IZSPLV), Torino, Italy

**Keywords:** *Chlamydia pecorum*, Whole genome sequencing, Plasticity zone, Polymorphic membrane protein, Plasmids, Chamois, Water buffalo

## Abstract

**Background:**

To date, whole genome sequencing has been performed mainly for isolates of *Chlamydia trachomatis*, *C. pneumoniae*, *C. psittaci* and *C. abortus*, but only a few isolates of *C. pecorum* have been entirely sequenced and this makes it difficult to understand its diversity and population structure. In this study the genome of two *C. pecorum* strains isolated from the lung of an Alpine chamois affected with pneumonia (isolate PV7855) and the brain of a water buffalo affected with meningoencephalomyelitis (isolate PV6959), were completely sequenced with MiSeq system (Illumina) and analyzed in their most polymorphic regions.

**Results:**

The genome length and GC content of the two isolates were found to be consistent with other *C. pecorum* isolates and the gene content of polymorphic membrane proteins and plasticity zone was found to be very similar. Some differences were observed in the phospholipase genes for both isolates and in the number of genes in the plasticity zone, such as the presence of some hypothetical proteins in PV6959, not present in any other genomes analyzed in this study. Interestingly, PV6959 possesses an extra *pmp* and has an incomplete tryptophan biosynthesis operon. Plasmids were detected in both isolates.

**Conclusions:**

Genome sequencing of the two *C. pecorum* strains did not reveal differences in length and GC content despite the origin from different animal species with different clinical disease. In the plasticity zone, the differences in the genes pattern might be related to the onset of specific symptoms or infection of specific hosts. The absence of a tryptophan biosynthesis pathway in PV6959 may suggest a strict relationship between *C. pecorum* and its host.

**Supplementary Information:**

The online version contains supplementary material available at 10.1186/s12864-022-08860-7.

## Background

*Chlamydia pecorum* is a Gram-negative obligate intracellular pathogen belonging to the *Chlamydiaceae* family, which includes organisms characterized by a biphasic cycle with a metabolically inert extracellular form, called elementary body (EB) and a metabolically active intracellular form, the reticulate body (RB) [1]. Its ability to replicate inside host cells is a key factor for the organism to remain hidden from the host immune response and cause persistent infections [2]. *C. pecorum* can infect a broad range of domestic and wild animals, including small and large ruminants (sheep, goats and cattle), horses, swine, birds and marsupials. It can cause polyarthritis, pneumonia, urogenital tract infections, abortion, conjunctivitis, mastitis, encephalomyelitis, enteritis, pleuritis and pericarditis [3, 4] which represent an economic concern to the farming industry globally, while only in some cases *C. pecorum* infections are asymptomatic [5].

Initially, the main method for typing chlamydiae was by using monoclonal and polyclonal antibodies [6], but with the development of molecular biology, new genotyping methods have been applied, including PCR-RFLP and sequencing of individual genes, e.g. *ompA* and *incA* [7]. In the last few years, the advent of whole genome sequencing (WGS) has allowed the analyses of the entire genome and specific regions and genes associated with virulence, tissue tropism and host range. In chlamydial species, WGS has enabled the comparison of gene content and synteny, as well as the identification of single-nucleotide polymorphisms (SNPs), detailed analysis of specific regions in the chlamydial genome where nucleotide variations and differences occur more frequently. Such regions are thought to be involved in differences in virulence, pathogenesis and niche specificity, and include the plasticity zone (PZ), the loci encoding the polymorphic membrane protein (Pmp) gene family, the tryptophan biosynthesis operon (trp), which is responsible for the tryptophan production and biotin biosynthesis gene region, which leads to biotin production [4]. Another feature of difference between chlamydial species and strains lies in the presence or absence of a plasmid, which has been linked to virulence and pathogenesis [8–10].

Currently 14 genomes of *C. pecorum* isolated from ruminant and swine livestock species and koalas are deposited in public databases, six of which are complete (Table 1). The aim of this study was to compare the full sequenced and analyzed genomes of two strains of *C. pecorum* isolated from a wild and a farmed ruminant, exhibiting different disease manifestations. PV7855 was isolated in 1996 from the lung of an Alpine chamois (*Rupicapra r. rupicapra*) found in the Adamello Brenta Park in the Alpine region of northern Italy and affected with pneumonia [11] belonging to a population that shared the grazing area with farmed ruminants (sheep and cattle). PV6959 was isolated from the brain of a farmed water buffalo (*Bubalus bubalis*) affected with meningoencephalomyelitis in a farm in Southern Italy [12] where several buffalo calves were affected with sudden depression, recumbency, paralysis of the limbs and amaurosis.

**Table 1:** General features of *C. pecorum* PV7855 and PV6959 compared with the reference strains

	***C. pecorum*** PV7855	***C. pecorum*** PV6959	***C. pecorum*** E58	***C. pecorum*** PV3056/3	***C. pecorum*** W73	***C. pecorum*** P787	***C. pecorum*** DBDeUG	***C. pecorum*** MC/MarsBar
Year of isolation	1996	1997	1940	1991	1989	1977	2010	2009
Country of origin	Italy	Italy	USA	Italy	Northern Ireland	Scotland	Australia	Australia
Source	Alpine chamois; lung	Water buffalo; brain	Calf; brain	Cow; cervical swab	Sheep; faeces	Sheep; synovial fluid	Koala; Urogenital tract	Koala; Urogenital tract
Disease pathotype	Pneumonia	meningoencephalomyelitis	Encephalomyelitis	Metritis	Asymptomatic/enteric	Polyarthritis	UTI	Chronic cystitis
Chromosome size (bp)	1,107,260	1,108,369	1,106,197	1,104,552	1,106,534	1,106,412	1,092,392	1,090,698
Plasmid size (bp)	7674	7676	NA	NA	7547	NA	7547	7547
% GC	41.1	41.2	41.1	41.1	41.1	41.1	41	41
Predicted CDS	990	987	1073	927	928	928	985	980
No. of tRNA genes	39	38	38	38	38	38	NA	NA
No. of rRNA operons	1	1	1	1	1	1	NA	NA
No. of sRNA molecules	3	3	3	3	3	3	NA	NA
No. of pmps	15	16	15	15	15	15	NA	NA

## Results

### Genome features

**Fig. 1:**
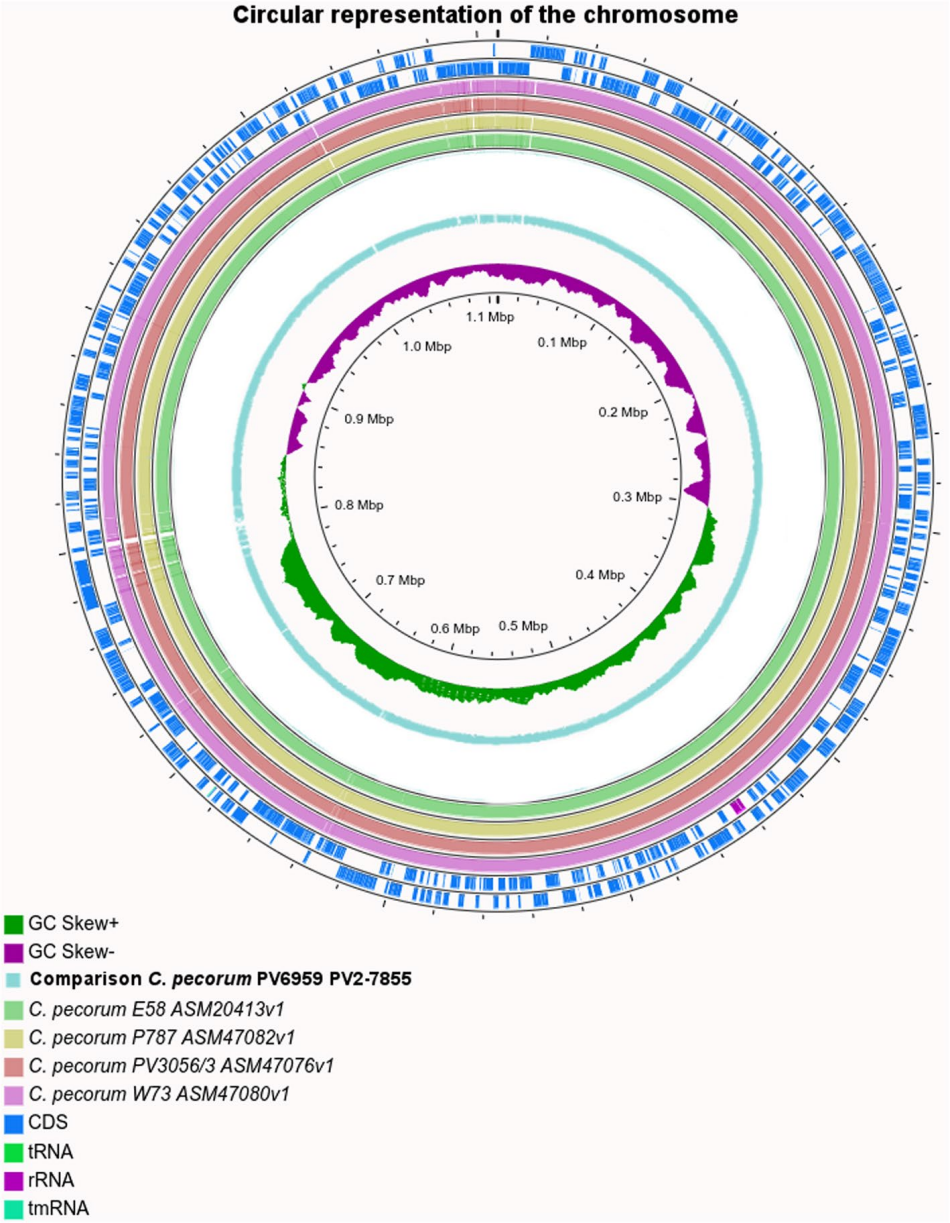
Circular representation of the comparison between chromosome of *C. pecorum* PV6959 and PV7855. Circles from the outside in represent the positions of protein-coding genes (blue), tRNA genes (green) and rRNA genes (pink) on the positive (circle 1), and negative (circle 2) strands respectively. It is shown the position of BLAST hits detected through blastn comparisons of PV6959 and PV7855 against W73 (circle 3), PV3056/3 (circle 4), P787 (circle 5), E58 (circle 6) with default settings; and GC skew (Circle 8). The image was generated with CGView Server

Sequencing of the genome of PV7855 resulted in a single circular chromosome of 1,107,260 bp with a read depth of 458x, while assembly of the genome of PV6959 resulted in two gaps and two chromosomal contigs which were closed by PCR and sequencing giving a total length of 1,108,369 bp and a read depth of 327x. The circular representation and the general features of these genomes compared with those previously sequenced from sheep, cows and koalas affected with different disease pathotypes are shown in Fig. 1 and Table 1.

The GC content of PV7855 is 41.1%, while for PV6959 is 41.2%. Moreover, PV7855 presents an additional tRNA gene (tRNA- Cys (aca)) compared to the other genomes considered in this study. The annotation identified 990 predicted CDSs (of which 261 are hypothetical proteins) in PV7855 and 987 predicted CDSs (of which 263 are hypothetical proteins) for PV6959 (Table 1).

Both isolates sequenced in this study harbour a plasmid (Additional file 1: Fig. S1): pCpecPV7855 and pCpecPV6959. The annotation of the two sequences revealed a similar structure to that of chlamydial plasmids with 8 CDSs predicted in total and a length of 7674 bp with a coverage of 1050x for pCpecPV7855 and a length of 7676 bp with a coverage of 171x for pCpecPV6959 and the GC content is 31.7% for both of them. The plasmids consist of the following genes: three hypothetical proteins, a virulence protein *pGP3-D*, a DNA helicase, two tyrosine recombinase (*XerC*) and a sporulation inhibition protein (*Soj*)*.* The two copies of *XerC* present in pCpecPV7855 are very different, with an amino acid identity of 38,43%.

**Fig. 2:**
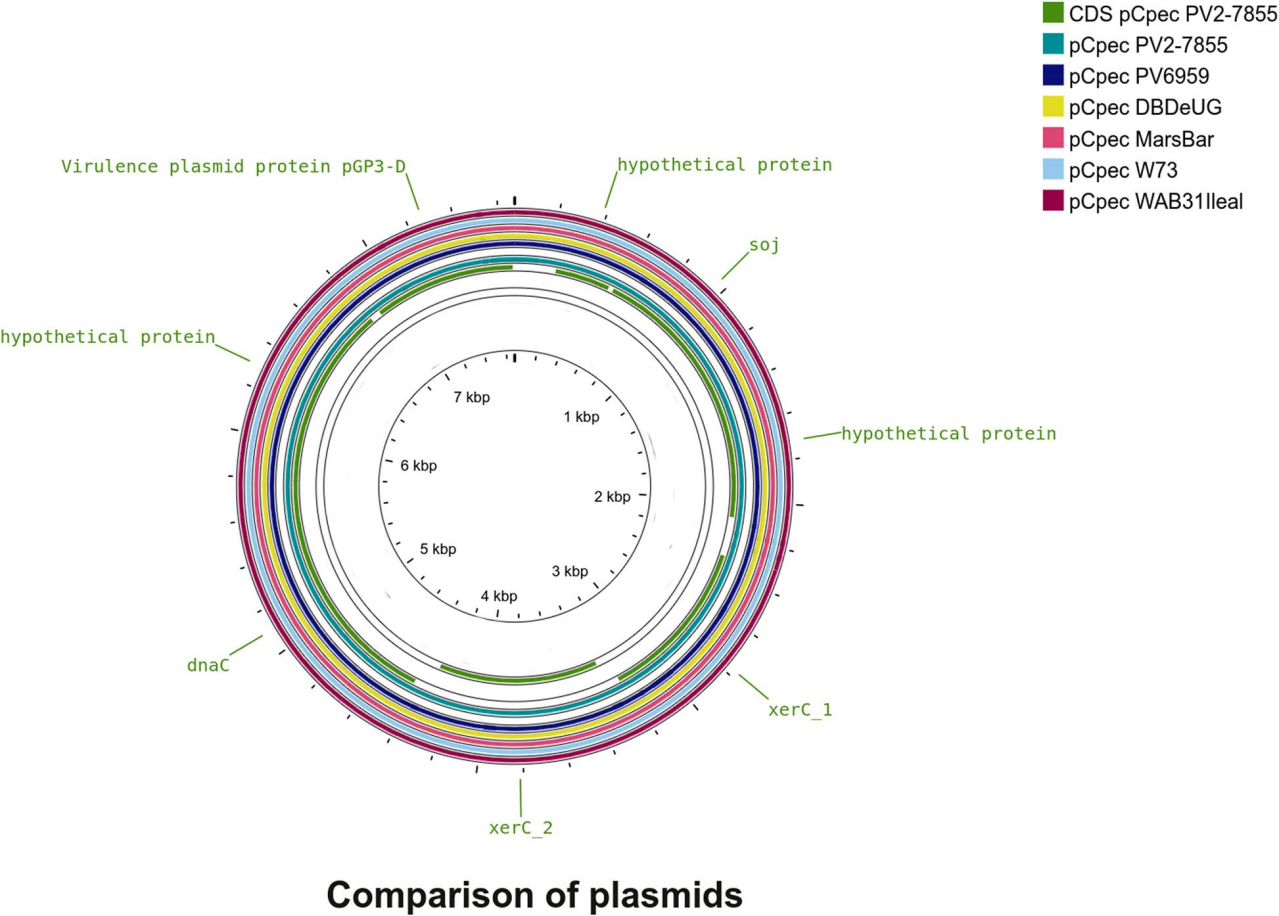
Circular representation of the comparison between plasmid pCpecPV6959 and pCpecPV7855. Circles from the outside inside represent the plasmids used for the comparison: pCpecWA/B31/Ileal (red), pCpecW73 (light blue), pCpecMC/MarsBar (pink), pCpecDBDeUG (yellow), pCpecPV6959 (blue) and pCpecPV7855 (aqua green). The image was generated with CGView Server

The circular representation of these plasmids compared with those belonging to genomes completely sequenced and included in this study and the plasmid of WA/B31/Ileal, are shown in Fig. 2.

### Phylogenetic and SNPs analysis

**Fig. 3:**
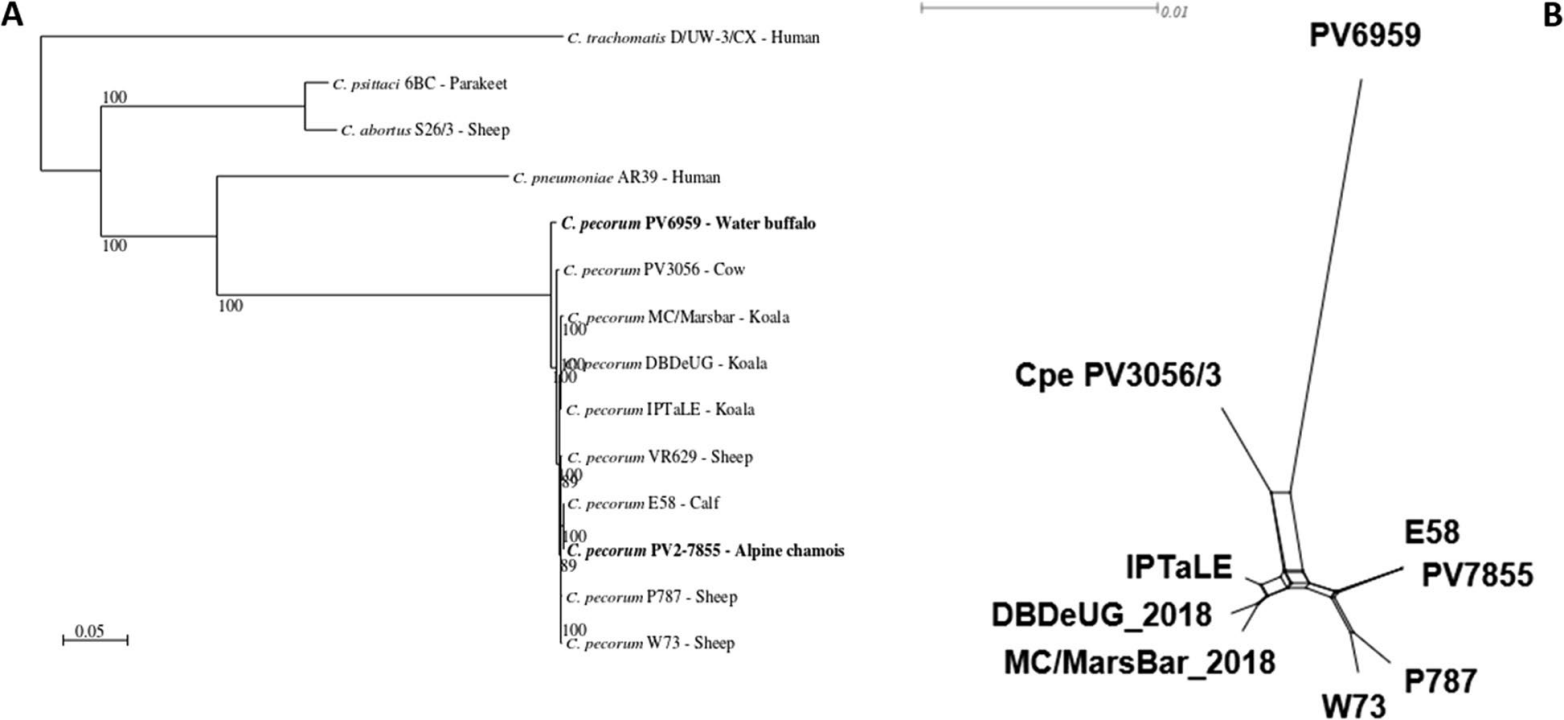
Phylogeny of selected *Chlamydia* species (**A**) and whole genome NeighborNet network analysis of *C pecorum* (**B**). **A** The box shows the relationship between the two sequenced *C. pecorum* genomes and others Chlamydia species (*C. trachomatis, C. psittaci, C. abortus* and *C. pneumoniae*). The maximum-likelihood tree was reconstructed using OrthoFinder v. 2.4.0 with modeltest-ng v 0.1.7 based on the nucleotide sequences of the identified single copy orthologs genes present in all organism (753). **B** The box shows the phylogenetic network of a whole genome sequence alignment of *C. pecorum*, where the PV6959 isolate is completely separate from other strains. The scale bar indicates the expected substitutions per site. The figure was generated using SplitsTree4

To determine the relationship between the two sequenced *C. pecorum* genomes and the other representative *Chlamydiaceae* genomes selected for comparative analysis, a phylogenetic tree was constructed based on orthologous genes (Fig. 3). A strain of *C. abortus*, *C. pneumoniae*, *C. psittaci* and *C. trachomatis* were used as outgroup. The phylogenetic reconstruction obtained from the 753 single copy orthologs genes present in all organism (evolutionary model: LG + I + G4) is highly supported (all the nodes are supported by 100 bootstraps, with the exception of two nodes supported by 89 bootstraps) and shows a tight clustering of the *C. pecorum* strains. Phylogenetic analysis revealed that PV6959 is clearly separate from the other strains, including PV7855 with 8664 non synonymous SNPs. In contrast PV7855 is very close to E58 with only 125 non synonymous SNPs. The three *C. pecorum* strains isolated from Australian koala clustered separately from the majority of ruminant strains.

Our NeighbourNet analysis (Fig. 3 and Additional file 1: Fig. S2) using the same genomes as our phylogenetic tree confirmed that PV7855 is most closely related to strain E58, while PV6959 is well separate.

Even though there is a wide similarity across the genomes (Table 2 and Fig. 3) of *C. pecorum*, there are a significant number of SNPs, which contribute to variation among the genomes and, interestingly, PV7855 has a very low number of SNPs compared to E58 (125). The analysis of SNPs revealed also a great difference between PV7855 and PV6959 (8664 non synonymous SNPs). When PV7855 is compared with the *C. pecorum* genomes considered in this study, it shows a lower number of total SNPs and non-synonymous SNPs (179 with W73, 188 with P787, 198 with DBDeUG, 191 with MC/MarsBar), as opposed to PV6959, which has more differences (746 with PV3056/3, 820 with E58, 830 with W73, 834 with P787, 818 with DBDeUG and 823 with MC/MarsBar).

**Table 2:** Snps and non synonymous SNPs between *C. pecorum* genomes considered in the study

Total SNPs and non synonymous SNPs in ***C. pecorum***. genomes	N° of total SNPs	Non synonymous SNPs
PV7855 vs. PV6959	23,985	8664
PV7855 vs. PV3056/3	15,198	6639
PV7855 vs. E58	192	125
PV7855 vs. W73	566	179
PV7855 vs. P787	540	188
PV7855 vs. DBDeUG	626	198
PV7855 vs. MC/MarsBar	625	191
PV6959 vs. PV3056/3	2929	746
PV6959 vs. E58	3207	820
PV6959 vs. W73	3250	830
PV6959 vs. P787	3234	834
PV6959 vs. DBDeUG	3171	818
PV6959 vs. MC/MarsBar	3198	823

### Comparative analysis

Whole genome comparisons (Additional File 1: Fig. S3) show that the two *C. pecorum* genomes sequenced in this study are highly conserved and syntenic and have similar gene content to each other and to the other genomes included in the analysis.

**Fig. 4:**
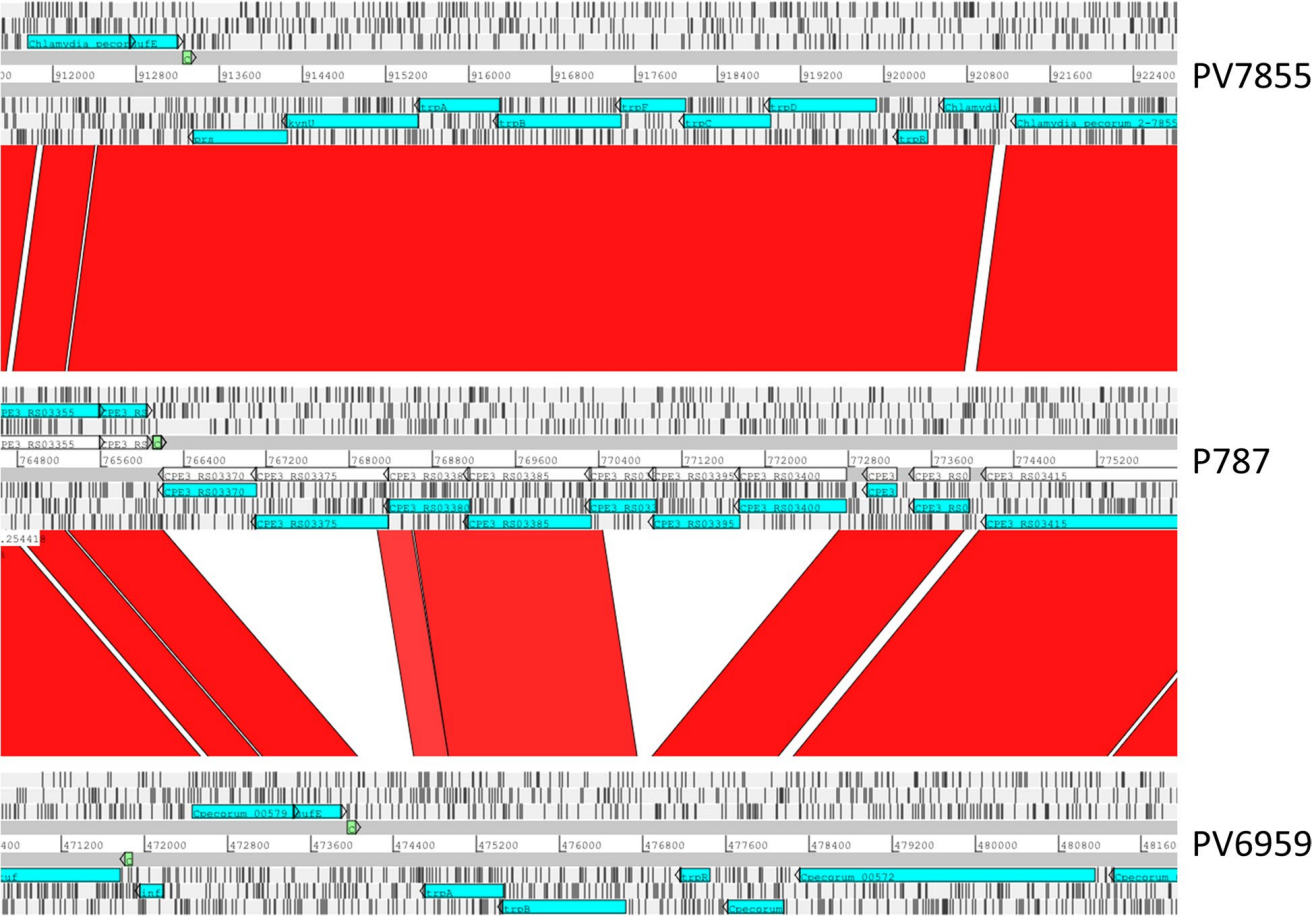
Comparative analysis of *trp* system*. Trp* system comparison between *C. pecorum* PV7855, P787 and PV6959 showing comparison of nucleotide matches between complete 6-frame translations (computed using Megablast blastn) using ACT. Grey bars represent the forward and reverse strands of DNA with CDSs marked as arrows. The scale is marked in base pairs. The red bars represent homology matches, the white ones represent the non-homology matches

Some regions reveal increased variability, including the PZ and the *pmps*. Some other regions are more conserved, such as the bioBFDA system, which is complete and highly homologous in both isolates and the trp operon of PV7855 which is complete and homologous to that found in the other genomes. Instead, in PV6959 the tryptophan synthase alpha chain (*trpA)*, tryptophan synthase beta chain (*trpB)* and tryptophan repressor (*trpR)* genes are present, while the *trpE/G* genes are missing (Fig. 4), as observed for other *C. pecorum* genomes studied to date [13], but we have also found that PV6959 lacks the *prsA*, *kynU*, and the *trpFCD* genes.

### Polymorphic membrane proteins

**Fig. 5:**
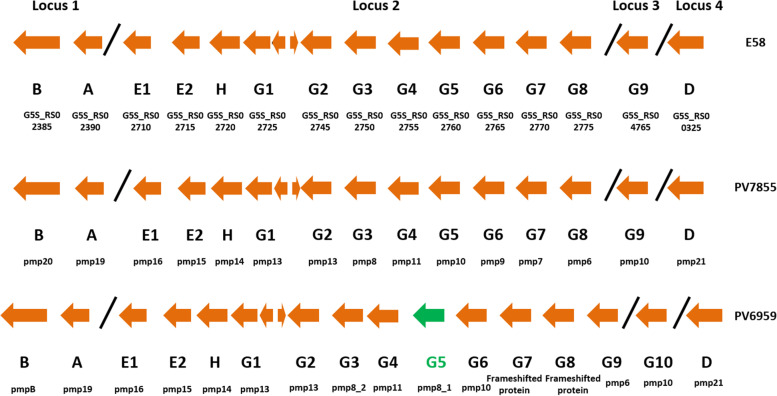
Polymorphic membrane proteins in *C. pecorum*. Genomic organization of *pmps* in *C. pecorum* with gene families (indicated under each block arrow). In PV6959 the extra *pmp* is indicated in green. The diagonal bars indicate the separation of the four different loci. Locus tag are available in Additional File 2 Table S1

A total of 15 *pmps*, which constitute one of the hotspots for SNPs accumulation, were observed in PV7855 and are characterized by one gene belonging to subtype B, one to subtype A, two to subtype E, one to subtype H, nine to subtype G and one to subtype D. The genomic organization of *pmps* of the genomes sequenced in this study is shown in Fig. 5, while the gene designations and annotations are listed in Additional File 2 Table S1. The comparison between pmps of PV7855 with the ones belonging to the genomes sequenced from ruminants, shows the degree of amino acid similarity between 87 and 100%. In contrast, pmp16 and pmp15 of PV7855 compared to the correspondent of P787 and W73 have a lower degree of amino acid similarity, of 79% in both cases.

The comparison of this region with the loci of *pmps* of DBDeUG and MC/MarsBar revealed a rather high amino acid similarity between 98 and 91%, except for pmp6 (Cpecorum_PV6959_00975; Chlamydia_pecorum_2-7855_00564) (82%).

In PV6959, however, we detected the presence of 16 *pmps*, indeed an extra *pmp* belonging to the G family was identified, *pmpG5* (Cpecorum_PV6959_00982). The region comprising the gene was amplified and sequenced with Sanger method and the consensus sequence was analyzed with BLAST showing 76% of nucleotide identity with P787, E58, W73, PV3056/3 and DBDeUG. The presence of two frameshifted pmps was observed in the same genome (corresponding to pmpG7 to pmpG8) while no frameshift was observed in the pmps of PV7855. The annotation identified two hypothetical proteins in both genomes, corresponding to *pmpG4* and *pmpG8* in PV7855 and *pmpG4* and *pmpG9* in PV6959. The pmps of PV6959 compared to those of the other strains are more variable in the amino acid sequence, especially the two pmps belonging to E family, pmp16 (Cpecorum_PV6959_00005) and pmp15 (Cpecorum_PV6959_00004), and the two pmps belonging to G family (pmp9 and pmp10). Indeed, when pmp16 and pmp15 are compared with the amino acid sequences of the correspondent of the genomes considered, the degree of identity is between 83% with E58 and 72% with P787 and W73. When we considered the pmps of DBDeUG and MC/MarsBar, we noticed there is a rather high similarity with PV6959. The lower identity is observed always in pmp16 (84%) and in pmp15 (82%).

We noticed that pmp6 appears as the pmp having the most differences in the amino acid sequence when it is compared with that of the other genomes analyzed. Indeed, in PV7855 its homology ranges between 78.22% (with PV6959) and 99.71% (with E58), while in PV6959 it ranges between 77.04% (with PV3056/3) and 87.97% (with P787).

### Plasticity zone

**Fig. 6:**
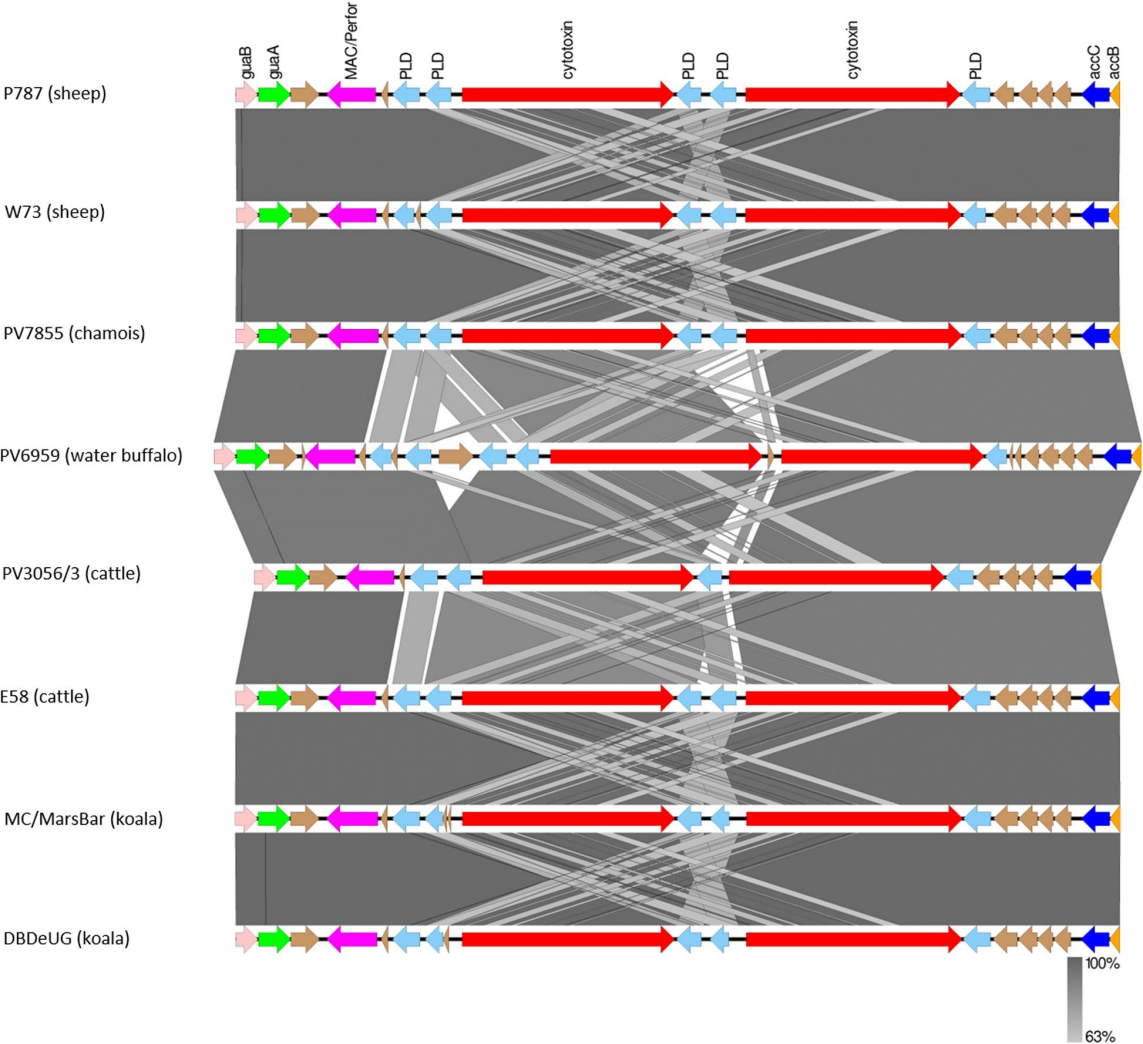
Visual representation of the genomic island of the plasticity zone in *C. pecorum*. Comparative analysis of the genes in the plasticity zone of *C. pecorum* PV7855 and PV6959. Comparison of the nucleotide matches (computed using blastn) between the genes *guaB* (pink) and *accB* (orange) in *C. pecorum* strains. The brown genes indicate hypothetical proteins. The orientation of coding sequences in the forward and reverse frames are indicated by the direction of the block arrows. The level of BLAST identity between the sequences is indicated by the degree of grey shading in the vertical bars. The figure was generated using EasyFig [14]

A genomic island, which is the most variable region in gene content and sequence, is the PZ, located near the terminus of replication. In *C. pecorum* strains, the PZ is generally around 42 kb in size and included between the acetyl-CoA-carboxylase genes (*accB* and *accC*) and the inosine-5′-monophosphate dehydrogenase (*guaAB*/*add*) genes. The two *C. pecorum* genomes sequenced in this study have PZs distinct from each other: the PZ of PV7855 is most similar in gene order to that of W73 with 18 genes, while in PV6959 we identified 24 genes, in particular a second set of *pld* genes upstream of the *tox* genes (Fig. 6). The comparison of our genomes with the others, all from ruminant hosts, resulted in a syntenic gene order and a high genetic relatedness. In contrast, while the gene order was syntenic with the PZs in koala derived *C. pecorum* genomes, the genetic relatedness was lower.

We identified 5 hypothetical proteins in PV7855 which have a degree of amino acid identity between 95 and 100% with the correspondent proteins in the genomes considered. The correspondent of Chlamydia_pecorum_2-7855_00297 in PV3056/3 and PV6959 represents the only exception with, respectively, the 62% and the 59% of amino acid similarity.

In PV6959, we identified 6 hypothetical proteins, one of which is located between the two set of *pld* genes. The degree of amino acid identity with the correspondent proteins in the genomes considered ranged between 99 and 97%.

In PV7855 we identified 4 copies of the *pld* genes and a gene labeled as cardiolipin synthase (*cls*), which is also part of the phospholipase family. In contrast, in PV6959 we identified three *pld* genes (*pld1*, *pld2* and *pld3*) plus two genes noted as cardiolipins (*cls1* and *cls2)* and the degree of amino acid homology are listed in Table 3. Searches against GenBank using BLAST confirmed their annotation as cardiolipins.

**Table 3:** Degree of amino acid similarity between pld and cardiolipin synthase in PV7855 and PV6959

Gene name	***C. pecorum*** E58vsPV7855	***C. pecorum*** PV3056/3vsPV7855	***C. pecorum*** W73vsPV7855	***C. pecorum*** P787vsPV7855	***C. pecorum*** E58vsPV6959	***C. pecorum*** PV3056/3vsPV6959	***C. pecorum*** W73vsPV6959	***C. pecorum*** P787vsPV6959
**PLD1**	100%	56.81%	93.04%	92.11%	95.93%	83.72%	95.63%	59.30%
**PLD2**	100%	NA	95.95%	94.69%	54.45%	NA	NA	NA
**PLD3**	100%	NA	NA	NA	59.15%	96.61%	58.55%	58.59%
**PLD4**	100%	80.04%	98.62%	48.39%	NA	NA	NA	NA
**CLS1**	99.28%	NA	95.04%	94.35%	NA	NA	NA	NA
**CLS2**	NA	NA	NA	NA	61.77%	NA	NA	NA

In both isolates sequenced in this study, we identified two copies of the cytotoxic genes. In PV7855, the similarity of amino acid sequence of *toxB* genes is lower when compared to the *toxB* of the isolates PV3056/3 (87.58–86.56%) and those of PV6959 (83.44–87.61%). In PV6959 the amino acid similarity of toxB is between 83 and 94% when compared to the genomes considered.

### Phylogenetic and comparative analysis of plasmids

**Fig. 7:**
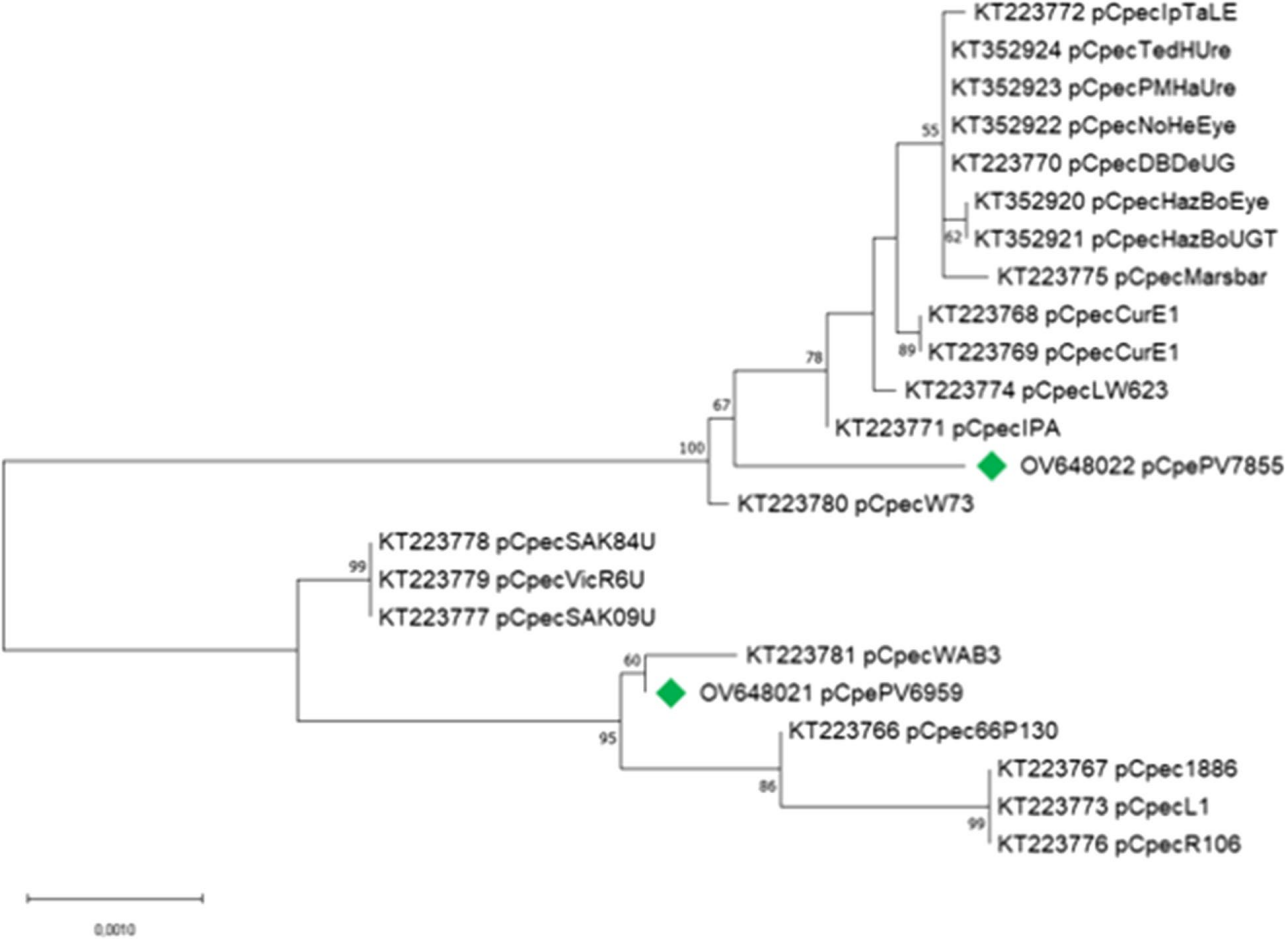
Phylogenetic analysis of plasmid sequences of *Chlamydia pecorum*. The maximum Likelihood tree was reconstructed using MEGA v. 11, using the Tamura 3 parameter model. The tree with the highest log likelihood (−11,062,28) is shown. The percentage of trees in which the associated taxa clustered together is shown next to the branches. The tree is drawn to scale, with branch lengths measured in the number of substitutions per site. This analysis involved 23 nucleotide sequences. Codon positions included were 1st + 2nd + 3rd + Noncoding. There was a total of 7803 positions in the final dataset

The phylogenetic analysis based on alignment of the *C. pecorum* plasmids listed in Table 4, revealed that pCpecPV6959 is most closely related to the plasmid from WA/B31/Ileal (Fig. 7), which was isolated from cattle, while the plasmid of PV7855 is well separate from all the others, in line with the core gene phylogeny. Moreover, the tree shows two separate lineages of the plasmids of *C. pecorum* isolates from Australian koala.

**Table 4:** General features of *C. pecorum* plasmids used for phylogenetic analysis

Plasmid ID (p***Cpec***)	Country of origin	Source	Disease pathotype	Length (bp)	% GC	Accession number
PV7855	Italy	Alpine chamois; lung	Pneumonia	7674	31.7	OV648022
PV6959	Italy	Water buffalo; brain	Meningoencephalomyelitis	7676	31.7	OV648021
L1	Austria	Pig; lung	Pneumonia	7548	31.7	KT223773
W73	Ireland	Sheep; faeces	Asymptomatic/enteric	7547	31.6	KT223780
66P130	USA	Cattle; faeces	No clinical disease	7548	31.7	KT223766
1886	Austria	Pig; lung	Pneumonia	7548	31.7	KT223767
Cur/E11/Rec	Australia	Sheep; rectum	No clinical disease	7547	31.6	KT223768
Cur/E19/Rec	Australia	Sheep; rectum	No clinical disease	7547	31.6	KT223769
DBDeUG	Australia	Koala; UGT	UGT infection	7547	31.5	KT223770
IPA	USA	Sheep; joint	Polyarthritis	7547	31.6	KT223771
IPTaLE	Australia	Koala; ocular	Conjunctivitis	7547	31.5	KT223772
LW623	USA	Cattle; brain	Encephalomyelitis	7547	31.6	KT223774
MC/MarsBar	Australia	Koala; UGT	Cystitis	7547	31.5	KT223775
R106	Austria	Pig; lung	Pneumonia	7548	31.7	KT223776
SA/K09/Ure	Australia	Koala; urethra	No clinical disease	7547	31.6	KT223777
SA/K84/Ure	Australia	Koala; urethra	No clinical disease	7547	31.6	KT223778
Vic/R6/UGT	Australia	Koala; UGT	No clinical disease	7547	31.6	KT223779
WA/B31/Ileal	Australia	Cattle; ileum	Encephalomyelitis	7548	31.7	KT223781
HazBoEye	Australia	Koala; ocular	Conjunctivitis	7547	31.8	KT352920
HazBoUGT	Australia	Koala; UGT	Conjunctivitis	7547	31.8	KT352921
NoHerEye	Australia	Koala; ocular	Conjunctivitis	7547	31.5	KT352922
TedHUre	Australia	Koala; urethra	Cystitis	7547	31.5	KT352923
PMHaUre	Australia	Koala; urethra	Cystitis	7547	31.5	KT352924

## Discussion

*C. pecorum* is recognized as one of the most widely distributed chlamydial species, with a wide host range that includes livestock (sheep, goats, cattle and swine) and wildlife [15]. Despite this, very few genome sequences of *C. pecorum* have been deposited and thus made available, which is a limiting factor for investigating genetic diversity in this chlamydial species.

In this study, we sequenced and examined the genomes of two *C. pecorum* isolates, PV7855 from an Alpine chamois, which is the only *C. pecorum* isolate fully sequenced from a wild ruminant to date and PV6959 from a water buffalo [11, 12]. The genomes of our isolates were compared to those of E58, PV3056/3, P787 and W73, all recovered from farmed ruminants [13, 16], which have been selected for analysis because neither *C. pecorum* sequences from wild ruminants nor from water buffaloes are available in sequence repositories. Furthermore, PV6959 was isolated from a water buffalo reared in a farm where the observed clinical signs, the seropositivity to *Chlamydia*, the histological findings, the detection of *C. pecorum* by immunofluorescence assay in the brain tissue and eventually the isolation of the organism in cell culture, confirmed the diagnosis of chlamydial meningoencephalomyelitis [12]. The two isolates were compared also versus the two genomes obtained from Australian koala (DBDeUG and MC/MarsBar) with regard to the two main variable regions, PZ and *pmps*.

The genome length and GC content of PV7855 and PV6959 isolates are similar to those of the other *C. pecorum* isolates deposited in the genome repositories. The *C. pecorum* genome, like other chlamydial species, has a conserved order and gene content [13] and an explanation for this could be that *Chlamydia* tried to reduce the exposure to frequent lateral gene transfer events, which can cause phenotypic modifications [17].

The phylogenetic analysis based on the orthologous genes shows a tight cluster of the *C. pecorum* strains, but interestingly, PV6959 appears to be well separate from the other *C. pecorum* strains considered in this analysis, while PV7855 is very close to E58. This confirm the data from sequence analysis and the number of SNPs, which demonstrate that PV7855 and E58 are very similar in genetic structure. PV7855 and PV6959 are both isolates from ruminants and, as already demonstrated [4], are well separate from *C. pecorum* isolates from Australian koala, maybe due to their different geographic origin and host preference. NeighborNet analysis gave further confirmation that PV7855 and E58 are evolutionarily close, in fact, the genome analysis revealed a high similarity (range from 97 to 100%) in the amino acid sequences of pmps, the PZ, the trp system and bioBFDA system, furthermore, they present a very low number of SNPs. To better clarify the phylogenetic distance between isolates from koala and wild ruminants more NGS analysis on different isolates are needed.

Firstly, we focused our analysis on the *pmp* loci, which are known to be one of the most polymorphic loci in the entire chlamydial genome. The members of the *pmps*, which are part of Type V Secretion System, are unique to chlamydial species [18] and possess a conserved domain structure that includes the C-terminal autotransporter beta-barrel domain, a central m-domain unique to this family of proteins and an N-terminal passenger domain that is involved in adhesion [19].

Pmps are involved in the adhesion to the host’s cell as autotransporter surface-exposed proteins, but are also involved in the immunopathogenesis of *Chlamydia* infections as potent antigenic proteins [20]. The number of *pmps* varies according to the chlamydial species, for example *C. pecorum* contains 15 *pmps*, which show similarity in domain structure and are divided in 6 subtypes labeled as: A, B, D, E, H and G, which has the highest number of *pmps* (nine) and the most variable genes among all the subtypes of *pmps*.

The number of *pmps* identified in PV7855 was 15, identical to the number identified in the isolates of *C. pecorum* deposited in the public databases. In contrast 16 *pmps* were identified in PV6959, one of which, Cpecorum_PV6959_00982 corresponding to pmpG5*,* is not present in any other whole genomes currently available. Even though PV6959 and E58 are characterized by the same disease pathotype and are evolutionarily very close (see NeighborNet analysis Fig. 3B), this Pmp is not present in E58. We can hypothesize that this protein is not involved in this specific pathogenesis, but more analyses from ruminants affected with the same disease are needed to better understand a possible correlation. The *pmp*G5 detected in PV6959 showed a low degree (76%) of nucleotide identity with P787, E58, W73, PV3056/3 and DBDeUG when it is run on BLAST and the Sanger-sequence of the region demonstrated that the gene is present and it is not an assembly or sequencing error.

PmpG6 is the most polymorphic Pmp in PV7855 and PV6959 with respect to the Pmps of the other *C. pecorum* isolates. This data supports other published studies showing that the Pmps belonging to the G family are those most subject to diversity in the sequence [8] and that the *pmps* are the most rapidly evolving genes in *C. pecorum* [4]. To date few studies have been carried out to investigate the function and the expression of *pmps* genes in *C. pecorum* [21].

The second region analyzed in our study is the PZ, where most of the studies focus on to evaluate the presence and/or absence of a range of established chlamydial virulence factors [22]. In the PZ of the *C. pecorum*, the number of *pld* genes can vary from 4 to 5, which is a matter not unique to *C. pecorum* [13], indeed also *C. trachomatis* and *C. muridarum* have a variable number of *pld* genes [23]. The PV7855 and PV6959 isolates each possess five copies of the phospholipase genes, of which one and two, respectively, are noted as cardiolipin. The gene annotated as cardiolipin synthas*e* in PV7855 corresponds to a *pld* gene in the other strains. Cardiolipin is present in three copies in *Escherichia coli* [24] and has been identified in *C. trachomatis,* where it appears to be expressed at 16 h after infection [25]. The role of *mac* and *pld* genes is still unclear but their respective functions appear to be linked [26] and they could be involved in the evasion of the host immune system and in facilitating entry and exit from the host cell. The *mac* genes, which encode membrane attack complex/perforin proteins, are not present in all Chlamydia species. Indeed, *mac* genes are present in *C. pecorum*, *C. felis*, *C. ibidis*, *C. muridarum*, *C. pneumoniae*, *C. psittaci*, *C. suis* and *C. trachomatis* [27]. In the two *C. pecorum* isolated in this study, the differences detected in Pld amino acid sequence compared with strains already present in repertoires, may suggest a role of these proteins in the difference of pathogenicity and virulence, but experimental studies are necessary to evaluate this hypothesis. Interestingly, PV6959 is the only strain, among those considered in this study, to have a hypothetical protein between the two *tox* genes instead of the two copy of *pld* genes. In contrast, among the mac/perforin domain, of the strains analyzed, there is a very high degree of amino acid identity, comprised between 95 and 99%.

The *toxB* cytotoxicity gene is present in a different number of copies depending on the chlamydial species, for example, there is only one copy in *C. psittaci*, *C. felis* and *C. caviae* and three copies in *C. muridarum*. In *C. pecorum*, *toxB* cytotoxicity gene is present in two copies, but its function is not yet fully known, it may contribute to the ability of the organism to switch from persistent infection to acute disease [13] and it could have different biological functions or a link to host specificity. Another peculiarity of PV6959 genome is the absence of the two *pld* genes between the two *toxB* genes. It represents the only case, between the genomes considered in this study, so the sequencing of more *C. pecorum* could be relevant also to understand the meaning of this aspect.

Genetic diversity also occurs in biotin biosynthesis, which leads the biotin production from pimeloyl-CoA, which shows significant variability among the different chlamydial species and is absent in some of them (*C. caviae*, *C. trachomatis* and *C. muridarum*) [22]. Biotin is involved in many central cell metabolism pathways and this could indicate its role in the host specificity [22]. In PV7855 and PV6959, it is present and highly homologous to the other systems belonging to the *C. pecorum* isolates.

*Chlamydia* species can be characterized for their ability to synthetize tryptophan and this ability depends on *trp* system, which is not complete in all chlamydial species. The *trp* system consists of *prs, kynU, trpD*, *trpF*, *trpC*, *trpA*, *trpB* and *trpR* and its aim, theoretically, is allowing the production of tryptophan from an anthranilate substrate [24]. In *C. pecorum* PV7855 sequenced in this study, *trp* system is almost intact, consisting of *trpD, trpF, trpC, trpA, trpB* and *trpR*, *prs* and *kynU*. This complement of genes would permit the production of tryptophan from the substrate anthranilate. However, this system does not allow the first step in the production of tryptophan, the conversion from chorismate to anthranilate, which would be catalyzed by *trpE*/*G*. It is hypothesized that the acquisition of anthranilate could be achieved through the capture of kynurenine from the host cell with a transport amino acid, *tyrP* [13]. In the isolate PV7855, *trp* system is complete and highly homologous to the one of the genomes considered in the comparison. In PV6959, however, the system is incomplete and in particular lacks the genes *prs*, *kynU*, *trpF*, *trpC* and *trpD.* The absence of these genes in a *C. pecorum* genome is detected in the present study for the first time, since *C. felis* and *C. caviae* represent two of the species, along with *C. pecorum*, where the *trp* system is complete. The system is absent in the PZ of *C. muridarum* and *C. psittaci* suggesting that the ability to synthesize *trp* de novo is not mandatory for the transmission or survival of these species [22]. PV6959 is likely unable to metabolize and produce tryptophan by itself, unless it manages to obtain indole in some other way.

Finally, an increasing number of recent studies have linked the chlamydial plasmids to pathogenesis [8–10]. Plasmids are also recognized as carriers of virulence factors and are almost ubiquitous in chlamydial species [9], thus, we also included plasmids in our analysis. Plasmids in *Chlamydia* are small, usually about 7.5 kbp in length, highly conserved, non-integrative and non-conjugative and they have been observed in many chlamydial species, including *C. pecorum* [8, 10]. They consist of non-coding RNA and eight open reading frames (ORF 1–8) [28] and their main role, in *C. trachomatis*, is the contribution in glycogen accumulation [29].

In this study, the plasmids found confirmed the presence of eight CDSs and a GC content in line as the other *C. pecorum* plasmids.

A comparison among the plasmids of PV6959, W73 and PV7855 shows slight differences. The W73 plasmid consists of eight genes, some noted as virulence factors that correspond to those noted as hypothetical proteins in plasmids of the isolates studied. In *Chlamydia* plasmid, CDSs 4 (the most polymorphic locus), 5 and 6 (the most conserved loci) are associated with virulence [30] and these data are confirmed in pCpecPV7855, which is composed of eight genes of which three are hypothetical proteins.

One limitation of our study was that, unfortunately, among the genomes selected for the chromosomal phylogeny only 3 strains (W73, DBDeUG and MC/MarsBar) have both a complete chromosome and plasmid. This meant our plasmid phylogeny is not directly comparable with the chromosomal phylogeny and highlights the lack of complete genomes for *C. pecorum* in public databases.

## Conclusions

The WGS and comparative analyses of PV7855 and PV6959 revealed that the two genomes are very similar in length and GC content and are highly preserved in the PZ and the *pmps*, the most polymorphic regions, in spite of the differences in the clinical manifestations of infections and the difference of host. Notwithstanding, in PV6959 an extra *pmp* was identified, confirmed by sequencing and genome analyses, but further studies will be necessary to clarify its role in host range. Some differences in the sequence and number of genes were also notice in the PZ, particularly in *pld* genes and hypothetical proteins. Since these genes are involved in virulence and evasion of immune response-system, their presence/absence might be related to the onset of specific symptoms or infection of specific host(s) and further studies are necessary to confirm or discard some of these hypotheses and to better understand their role.

Concerning the absence of a tryptophan biosynthesis pathway in PV6959, the host cell might provide tryptophan supplementation to *Chlamydia*. Tryptophan is an essential amino acid required for development of all *Chlamydia* species and their dependency on the host suggests a strict relationship between *C. pecorum* and its host. Even more interestingly, tryptophan metabolism has been implicated in chlamydial persistence and tissue tropism.

We consider it would be useful to investigate whether the genomic differences that we observed in our isolates are present also in others from ruminants with the same pathology. In addition, a genomic comparison with isolates from non-ruminant species would allow an investigation on whether the differences noted are linked to the pathology rather than to the host species.

## Methods

### Strain features, propagation on cell cultures and EB purification

Strain PV7855 was isolated in 1996 from the lung of an Alpine chamois found in the Adamello Brenta Park in the Alpine region of northern Italy and affected with pneumonia [11]. The isolate was extracted from a tissue suspension with a mortar and sterile quartz powder and then centrifuged at 108 x *g* for 10 minutes. The supernatant was inoculated on LLC-MK2 cells monolayer which were cultured in EMEM (*Eagle’s Minimum Essential Medium*) with the supplement of L-glutamine 1% (v/v), gentamicin 10 mg/L, vancomycin 10 mg/L, glucose 720 mg/L (SigmaAldrich), and inactivated Fetal Calf Serum (FCS) at 10% (Gibco®, Life Technologies) and incubated at 37 °C and 5% CO_2_. L-glutamine, gentamicin and vancomycin were supplied by AppliChem. Infection was verified by immunofluorescence with a monoclonal antibody (Merifluor® Chlamydia) conjugated with fluorescein specific for the lipopolysaccharide antigen of *Chlamydia*. The cell suspension was then recovered and the elementary bodies (EBs) were purified [31].

The same procedure was used for the strain PV6959, isolated from the brain of a farmed water buffalo affected with meningoencephalomyelitis in a farm in Southern Italy [12]. The supernatant was inoculated onto a McCoy cells monolayer and the infection was confirmed by real-time PCR. The DNA was amplified using primers and probe specific for the *ompA* gene of *C. pecorum* at a concentration of 0.6 μM and 0.25 μM, respectively and GoTaq® Probe qPCR Master Mix (Promega).

The EBs of both isolates were purified according to the protocol of Fukushi et al. [31] and resuspended in 200 μl of transport medium for *Chlamydia* SPG (Sucrose Phosphate Glutamate).

DNA was extracted from EB using a NucleoSpin® Tissue kit (Macherey-Nagel) according to manufacturer’s instructions.

### Whole genome sequencing and genome assembly

The DNA extracted from the isolates, was used to prepare the genomic libraries using the Nextera XT kit (Illumina), quantified with KAPA SYBR FAST Universal qPCR Kit (Roche) and sequenced on a Miseq system (Illumina) in a 2x250bp run generating paired-end reads.

The quality of the reads obtained was assessed using FastQC [32], while the presence of any contaminants was checked through the Kraken software [33] and an in-house pipeline based on the blast of the reads with subsequent analysis using the MEGAN software [34].

The reads were separate from those of the host, following the “blobology” bioinformatics pipeline [35] and then assembled using SPAdes v. 3.10 [36]. Possible assembled contig joinings were evaluated using Bandage [37], tested by PCR and then sequenced for confirmation. The primers used were designed on non-repeated regions of the ends of the contig and their specificity was verified by BLAST.

The PCR products were purified with the NucleoSpin® Gel and PCR Clean-up kit (Macherey-Nagel) and sequenced by the Sanger method. The electropherograms obtained were analyzed using the BioEdit software v.7.2.5 and the sequences obtained were compared with the previously assemblies and manually inserted into the junction points of the contigs. The assembly of the reads and the visualization of the assembly graph also allowed the identification of a plasmid for each of the isolates.

### Phylogenetic and comparative analysis

Genome assemblies were annotated using Prokka v. 1.12 [38] with default parameters and the following genomes sequenced were used in phylogenetic analysis with the two *C. pecorum* sequenced in this study, using OrthoFinder v.2.4.0 [39]: *C. trachomatis* D/UW-3/CX (accession number: NC_000117.1), *C. psittaci* 6 BC (accession number: NC_017287), *C. abortus* S26/3 (accession number: NC_004552), *C. pecorum* W73 (accession number: NC_022440), *C. pecorum* P787 (accession number: NC_022441), *C. pecorum* E58 (accession number: NC_015408), *C. pecorum* PV3056/3 (accession number: NC_022439), and *C. pneumoniae* AR39 (accession number: AE002161 AE002164-AE002268). The identified single copy orthologs genes present in all organism (753) were aligned with MUSCLE v3.8.31 (default settings) [40] and hypervariable regions were removed with Gblocks v0.91b (default settings) [41]. The sequences were then concatenated with an in-house Perl script. In order to infer the phylogeny, the evolutionary model was chosen according to the best AIC score obtained with modeltest-ng v0.1.7 (default settings) [42] and RAxML v.8.2.11 was run (100 bootstraps, −m PROTGAMMILG, −× 1234, −p 123) [43].

Artemis Comparison Tool (ACT) software v.17.0.1 [44] was used to compare each genome with others present in NCBI and selected on the basis of assembly level.

The amino acid sequences of the genes of the PZ, the *pmps* cluster, the *bioBFDA* system and *trp* system, were compared by ClustalW to evaluate the degree of homology with the corresponding sequences of the other genomes.

The SNPs detected in the comparison of genomes, including the number of synonymous and non-synonymous substitutions, were identified with the Purple pipeline [45].

The presence of an additional *pmp* was checked bioinformatically by mapping the reads to the coding region of the *pmp* and the flanking regions (1000 nt upstream and 1000 nt downstream) using Bowtie2. The mapped reads were sorted and indexed using Samtools and sequence coverage observed graphically using BamView (Artemis release: 18.1.0) [46]. In addition, a walking PCR was performed to confirm the presence of the gene, the primers (Table 5) and the thermic cycle were designed ad hoc. The PCR products were excised from agarose gel and sequenced with Sanger method. The obtained sequences were concatenated with BioEdit Software v. 7.2.5 and the consensus sequence analyzed with BLAST tool (NCBI, http://blast.ncbi.nlm.nih.gov/).

**Table 5:** Primers used for the sequencing of the extra pmp in PV6959

	FW	REV	Amplicon size
6959_1	GTGCATTGGGAAAGCAATTTGA	CACTACCTGTCGTGGAGTTGT	723 bp
6959_2	GGGAACAATAGCGATGGTGC	GTTTCTAGGGTTGTTCCTGCATT	677 bp
6959_3	GACGGAGTGACTCTACAAGCA	AGATGGCGGAATCCCTGTT	668 bp
6959_4	GGAAATCATAGCAGCTTGGGA	AGGGTTAATTTCGTGTAGGTGG	576 bp
6959_5	CTCCCTATCGCTGCAAGT	TGAGACATTTCGGGTCGTG	422 bp

Whole genome sequences were aligned using MAFFT v7.450 [47] with a FFT-NS-i strategy. Phylogenetic network analysis for inferring evolutionary relationships between the MAFFT aligned genome species and strains was performed using SplitsTree v4.17.1 [48].

The evolutionary history was inferred by using the Maximum Likelihood method and Tamura 3-parameter model. Initial tree(s) for the heuristic search were obtained automatically by applying Neighbor-Join and BioNJ algorithms to a matrix of pairwise distances estimated using the Tamura 3 parameter model, and then selecting the topology with superior log likelihood value. The tree is drawn to scale, with branch lengths measured in the number of substitutions per site. This analysis involved 23 nucleotide sequences. Codon positions included were 1st + 2nd + 3rd + Noncoding. There was a total of 7803 positions in the final dataset. Evolutionary analyses were conducted in MEGA11 v. 11..

### Nucleotide sequence accession numbers

Genome sequences of *C. pecorum* strains PV7855 and PV6959 have been deposited in ENA GenBank under the Bioproject numbers PRJEB25743 and PRJEB25774, respectively.

## Supplementary Information

**Additional file 1: Figure S1.** Circular representation of the chromosome of the plasmid of *C. pecorum* PV7855 and PV6959. **Figure S2.** Whole genome NeighborNet network analysis. **Figure S3.** Comparative analysis of *C. pecorum*.

**Additional file 2: Table S1.** Locus designations and annotations of pmps of PV7855 and PV6959.

## Data Availability

The strains will be available in the IZSLER veterinary biobank (http://www.ibvr.org). Chromosome assemblies are available with the Nucleotide ID GCA_900464915 and GCA_927312825 for PV7855 and PV6959 strains, respectively. Chromosome assemblies of plasmid are available with the Nucleotide IDOV648022 and OV648021 for pCpecPV7855 and pCpecPV6959, respectively.

## Abbreviations

WGS: Whole Genome Sequencing
PZ: Plasticity Zone
PMPS: Polymorphic membrane proteins
EB: Elementary body
RB: Reticulate body
BioBFDA: Biotin synthesis system
Trp operon: Tryptophan synthesis operon
