# Supplementary figures and images for: Comparative analysis of two genomes of Chlamydia pecorum isolates from an Alpine chamois and a water buffalo
Source: BMC Genomics. 2022 Sep 10;23:645. doi: 10.1186/s12864-022-08860-7 (PMC9464383; doi:10.1186/s12864-022-08860-7)

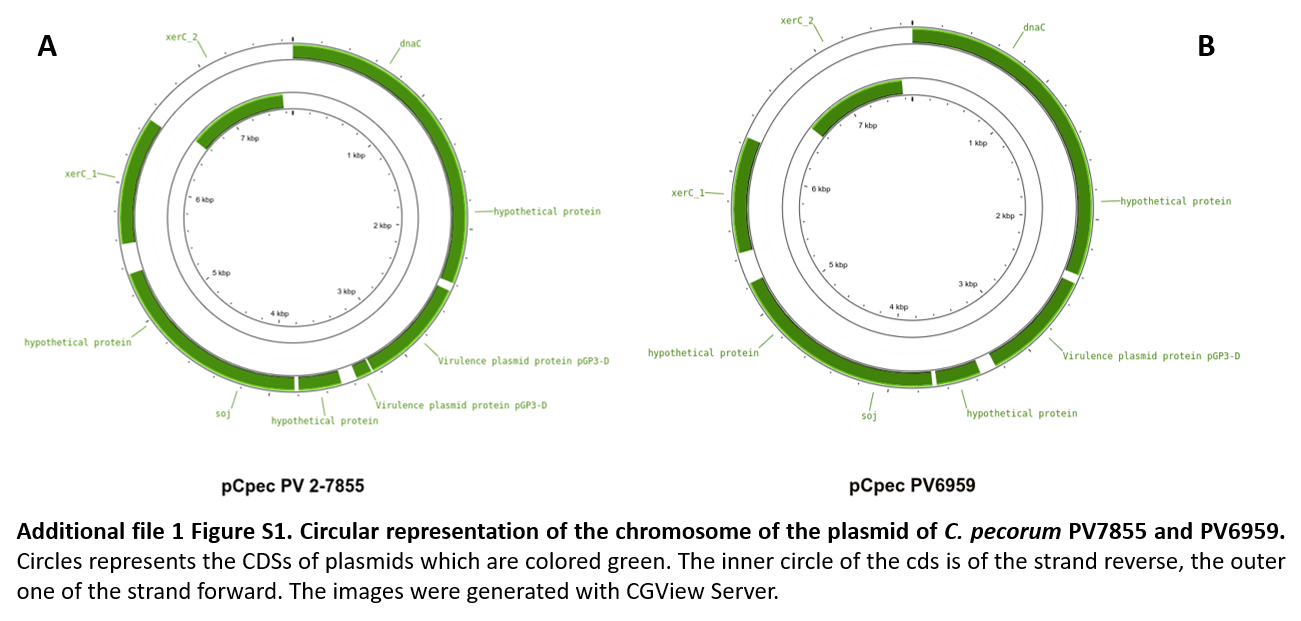

Supplement: Supplementary file 1 — Additional file 1: Figure S1. Circular representation of the chromosome of the plasmid of C. pecorum PV7855 and PV6959. Figure S2. Whole genome NeighborNet network analysis. Figure S3. Comparative analysis of C. pecorum. [file 12864_2022_8860_MOESM1_ESM.zip › AdditionalFile1FigureS1.png]

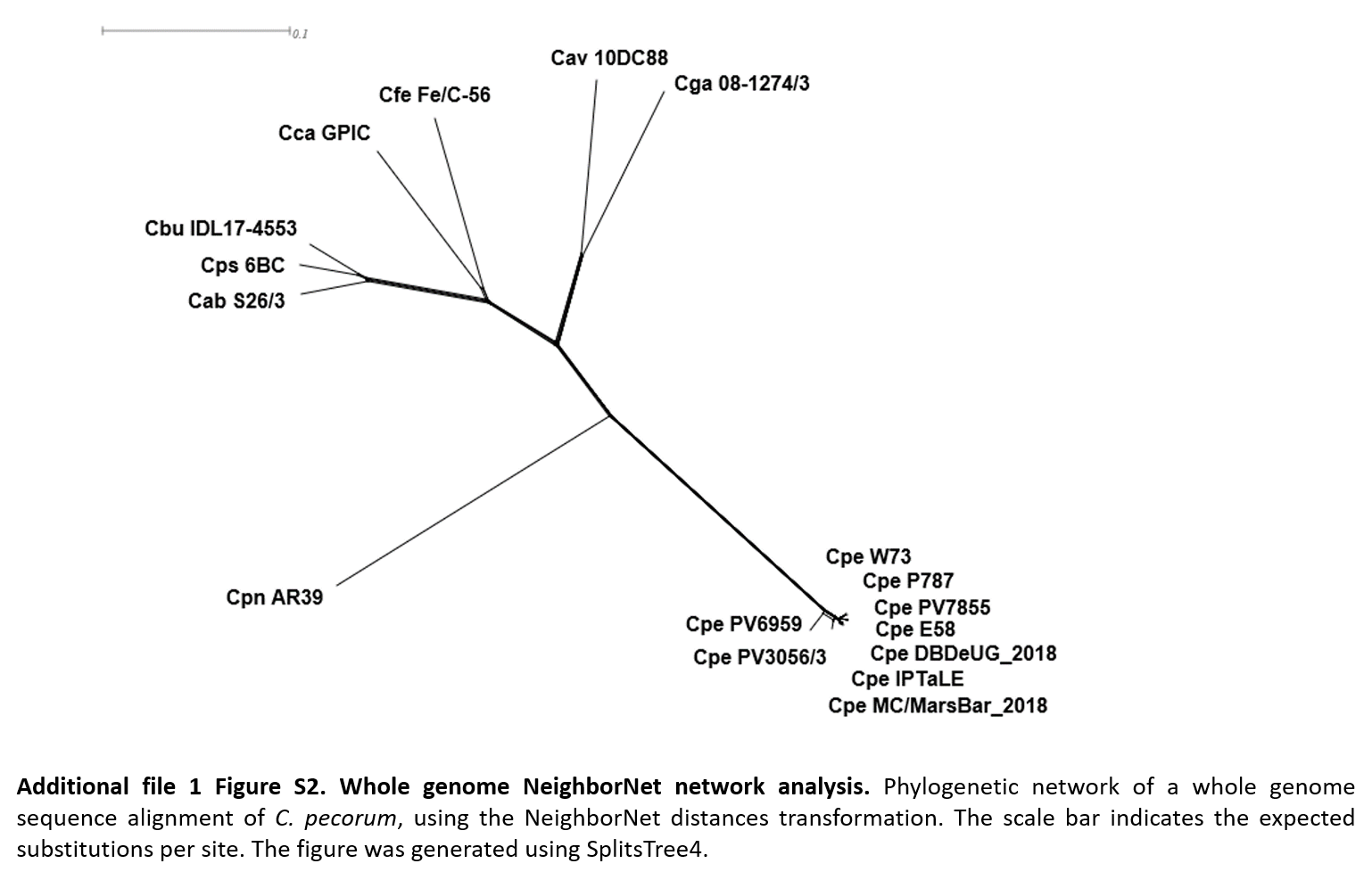

Supplement: Supplementary file 1 — Additional file 1: Figure S1. Circular representation of the chromosome of the plasmid of C. pecorum PV7855 and PV6959. Figure S2. Whole genome NeighborNet network analysis. Figure S3. Comparative analysis of C. pecorum. [file 12864_2022_8860_MOESM1_ESM.zip › AdditionalFile1FigureS2.png]

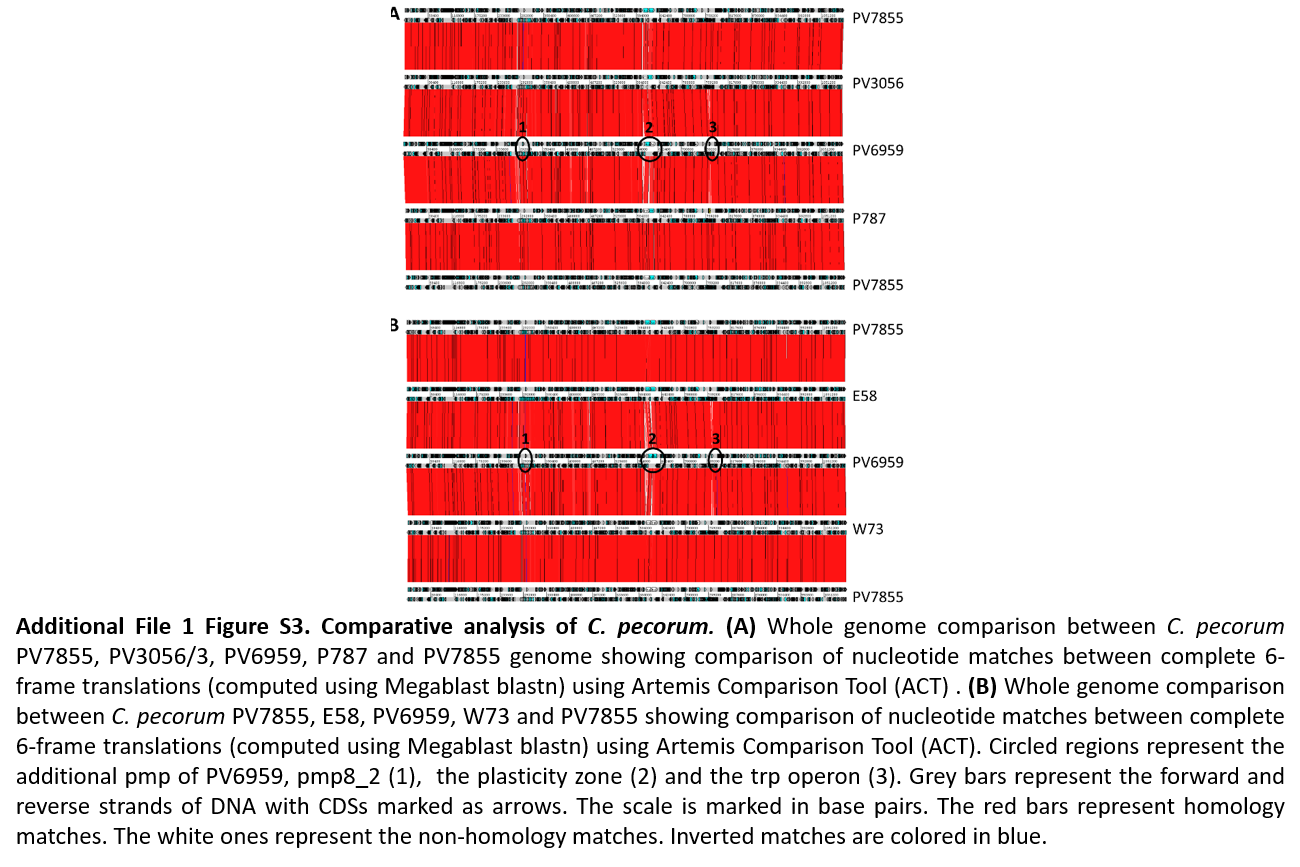

Supplement: Supplementary file 1 — Additional file 1: Figure S1. Circular representation of the chromosome of the plasmid of C. pecorum PV7855 and PV6959. Figure S2. Whole genome NeighborNet network analysis. Figure S3. Comparative analysis of C. pecorum. [file 12864_2022_8860_MOESM1_ESM.zip › AdditionalFile1FigureS3.png]

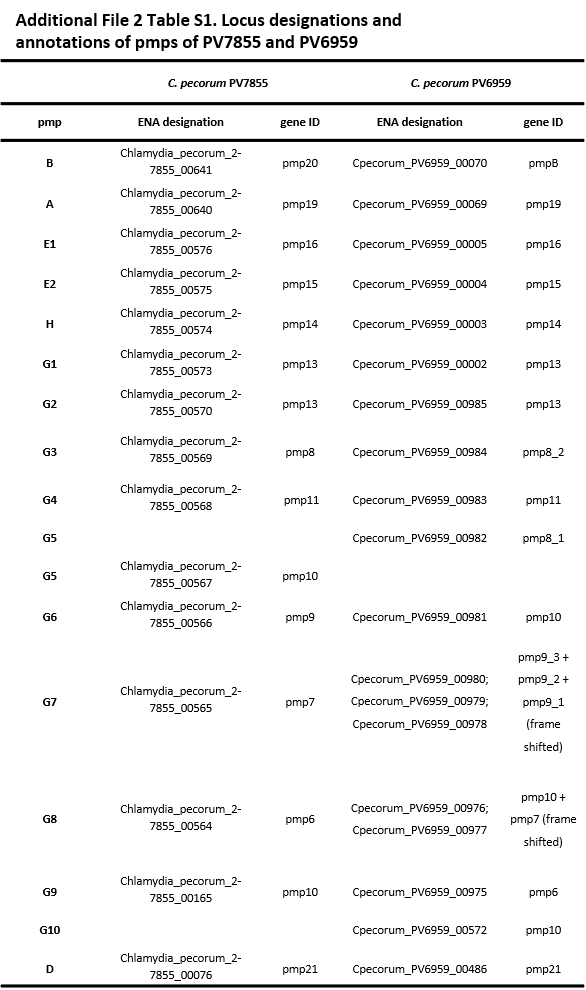

Supplement: Supplementary file 2 — Additional file 2: Table S1. Locus designations and annotations of pmps of PV7855 and PV6959. [file 12864_2022_8860_MOESM2_ESM.png]
